# Engineering *S. equi* subsp. *zooepidemicus* towards concurrent production of hyaluronic acid and chondroitin biopolymers of biomedical interest

**DOI:** 10.1186/s13568-017-0364-7

**Published:** 2017-03-14

**Authors:** Donatella Cimini, Ileana Dello Iacono, Elisabetta Carlino, Rosario Finamore, Odile F. Restaino, Paola Diana, Emiliano Bedini, Chiara Schiraldi

**Affiliations:** 1 Department of Experimental Medicine, University of Campania Luigi Vanvitelli (ex Second University of Naples), via de Crecchio 7, 80138 Naples, Italy; 2 Department of Chemical Sciences, University of Naples “Federico II”, Complesso Universitario Monte S. Angelo, via Cinthia, 4, 80126 Naples, Italy

**Keywords:** Chondroitin, Hyaluronic acid, Co-production, *Streptococcus equi* subsp. *zooepidemicus*

## Abstract

Glycosaminoglycans, such as hyaluronic acid and chondroitin sulphate, are not only more and more required as main ingredients in cosmeceutical and nutraceutical preparations, but also as active principles in medical devices and pharmaceutical products. However, while biotechnological production of hyaluronic acid is industrially established through fermentation of *Streptococcus* spp. and recently *Bacillus subtilis,* biotechnological chondroitin is not yet on the market. A non-hemolytic and hyaluronidase negative *S. equi* subsp. *zooepidemicus* mutant strain was engineered in this work by the addition of two *E. coli* K4 genes, namely *kfoA* and *kfoC,* involved in the biosynthesis of chondroitin-like polysaccharide. Chondroitin is the precursor of chondroitin sulphate, a nutraceutical present on the market as anti-arthritic drug, that is lately being studied for its intrinsic bioactivity. In small scale bioreactor batch experiments the production of about 1.46 ± 0.38 g/L hyaluronic acid and 300 ± 28 mg/L of chondroitin with an average molecular weight of 1750 and 25 kDa, respectively, was demonstrated, providing an approach to the concurrent production of both biopolymers in a single fermentation.

**Electronic supplementary material:**

The online version of this article (doi:10.1186/s13568-017-0364-7) contains supplementary material, which is available to authorized users.

## Introduction

Hyaluronic acid (HA) and chondroitin sulphate (CS) are important glycosaminoglycans (GAG) found in the extracellular matrix of vertebrates, and, although their applications in the biomedical field are quite diverse, growing interest towards both these compounds has indeed been observed in the last decade (Liu et al. 2011; De Angelis 2012). Due to its multiple properties including a high biocompatibility and lack of immunogenicity, HA is a heavily researched molecule with applications in cosmetics and viscosurgery, healing and regeneration of wounds and lately investigated as a drug delivery agent (Maytin 2016; Tripodo et al. 2015; Moscovici 2015). CS on the other hand, is a nutraceutical marketed as anti-arthritic drug, and also an active pharmaceutical ingredient (API) in oral formulations for osteoarthritis (Henrotin et al. 2010); studies towards other interesting applications such as the production of biomaterials for tissue engineering are also under development (Christiani et al. 2016; Bishnoi et al. 2016). Moreover the combination of both HA and CS was shown to be effective for the treatment of urinary infections (Lazzeri et al. 2016; Ciani et al. 2016; Cervigni et al. 2016), cartilage repair (Henson et al. 2012), and early symptomatic knee osteoarthritis (Galluccio et al. 2015).

HA was the first GAG produced by fermentation, and more than 90% of HA present on the market is obtained from group C *Streptococcus zooepidemicus*, as well as more recently from *Bacillus subtilis* (de Olivera et al. 2016). K4 and K5 polysaccharides are consolidated precursors of chondroitin and heparin; however, CS is still obtained from limited and potentially harmful, animal sources such as chicken keel, shark fins, and swine or bovine trachea. Several efforts have been made to use *E. coli* K4, or its biosynthetic machinery in a different background, to generate cell factories for the production of chondroitin, and encouraging results were obtained (Cimini et al. 2013, 2015; He et al. 2015; Jin et al. 2016). The reason lies in the fact that the capsular polysaccharide of this uropathogenic strain, composed of alternating residues of *N*-acetylgalactosamine (GalNAc) and glucuronic acid (GlcA), has a structure that closely resembles the backbone of CS. Differences regard the presence of a fructose side branch on the GlcA residues and the absence of sulphate groups. Biotechnological chondroitin (BC), produced from recombinant *E. coli* K4 (Cimini et al. 2013), was evaluated in comparison with CS showing a higher reduction of the inflammatory response in IL-1b treated chondrocytes and the enhancement of their proliferation and phenotype preservation (Stellavato et al. 2016). Furthermore, the chemical conversion of biotechnological chondroitin into CS by fructose branches cleavage and selective sulphation was recently accomplished (Bedini et al. 2011).

However, the gram− *E. coli* K4 releases in the medium also lipopolysaccharides that are the main product contaminants and need to be removed during the purification strategy. For this reason we focused on the use of a gram+ strain belonging to a genus that is widely used for the production of hyaluronan (Liu et al. 2011).

The gene cluster responsible for the biosynthesis of the K4 polysaccharide is composed of three regions, two of which (1 and 3) are conserved among group II *E. coli* strains and dedicated to the transport and secretion of the CPS. The central region, region 2, includes genes involved in the synthesis of the serotype specific polysaccharide, and in polymer assembly and fructosylation. Among the known gene functions *kfoC* codes for chondroitin polymerase (Ninomiya et al. 2002), a bifunctional enzyme that transfers nucleotide sugars to the growing end of the polysaccharide, and *kfoA* codes for an epimerase that converts *N*-acetylglucosamine (GlcNAc) into GalNAc. In the present work the *E. coli* K4 genes, *kfoC* and *kfoA*, were expressed in a previously obtained non hemolytic, hyaluronidase negative *S. equi* subsp. *zooepidemicus BA06* host (Schiraldi et al. 2010) to implement its UDP-biosynthesis pathway and polymer assembly machinery for the production of chondroitin, thereby generating a cell factory for the obtainment of both biopolymers in a single fermentation event.

## Materials and methods

### Bacterial strains

*Lactococcus lactis* NZ900 was used as an intermediate host for plasmid construction. The mutant strain (hyaluronidase-free and haemolytic negative) *S. equi* subsp. *zooepidemicus BA06* strain was obtained as previously described (Schiraldi et al. 2010). Plasmid pNZ8148 was obtained from Nizo (Netherlands).

### Materials

Genomic DNA and plasmid DNA were isolated using Qiagen DNeasy kit, Qiagen miniprep kit, (Qiagen, Valencia, CA) respectively according to the manufacturer’s instructions. Restriction endonuclease digestions, DNA ligations, agarose gel electrophoresis were performed using standard techniques (Sambrook and Russel 2001).

### Construction of the kfoA and kfoC overexpressing strains

The *kfoA* and *kfoC* genes were amplified from *E. coli* K4 genomic DNA. *kfoA* was amplified with primers kfoA_Up: 5′- GGACGGTGCCATGGATGAATATATTAGTTACAGGTGGAG-3′containing the *Nco*I recognition site, and KfoA_Dw 5′-TCTCTAGACGCGGATCCTTAAATATAACCATTTGGGTTTTTCA-3′ containing the *Bam*HI and *Xba*I restriction sites. *kfoC* was amplified with primers kfoC_ Up 5′-CGGGATCCAGTGAGGAGTTACTGATGAGTATTCTTAATCAAG-3′ and kfoC_Dw 5′-GAGCTCTTATAAATCATTCTCTATTTTTTCC-3′, containing the *Bam*HI and *Sac*I restriction sites respectively. Polymerase chain reaction (PCR) was carried out with Expand High fidelity PCR System (Roche, Monza. Italy) according to the manufacturer’s protocol. DNA fragments were recovered from agarose gels using the Qiaquick gel extraction kit (Qiagen, Valencia, CA). The *kfoA* and *kfoC* genes were sequentially cloned into plasmid pNZ8148 using *L. lactis* as intermediate host to improve transformation efficiency (Additional file 1).

Efficiency of sub-cloning was verified by performing double digestions with *Bam*HI and *Sac*I on the recombinant plasmid extracted from colonies of *L. lactis* growing on selective medium. Restriction endonucleases were purchased from New England Biolabs and ligases were purchased from Invitrogen (Carlsband, CA). Nucleotide sequencing of all PCR fragments cloned was carried out at BMR Genomics (Padova, Italy) to check whether any mutation was introduced.

pNZ8148*kfoAkfoC* was isolated from *L. lactis*-pNZ8148*kfoAkfoC* and electroporated into *S. equi* subsp. *zooepidemicus BA06* slightly changing the protocol described by Marcellin et al. (2010). Briefly, cells were grown on THY supplemented with 2 M glycine. About 250 mL of fresh medium were inoculated with an o/n pre-culture and grown for 3 h at 37 °C until OD_530_ reached a value of about 0.6. Prior to harvesting, 0.2 mg/mL of hyaluronidase (H3506, Sigma-Aldrich) were added to the broth and incubated for 30 min at the same temperature. After centrifugation the pellet was resuspended in 0.5 M sucrose and washed twice; finally it was resuspended in about 600 μL of the same solution. The recombinant plasmid pNZ8148*kfoAkfoC* was added to the ice cold cell aliquot and electroporation was carried out on a Biorad Bio-Rad Gene Pulser (2 mm cuvettes, 2.0 kV, 200 Ω, 25 μF) according to the protocol suggested by the manufacturer. Selection was performed on THY supplemented with 5 μg/mL of chloramphenicol. Plates were incubated o/n at 37 °C.

### Shake flask experiments

All cultivations were conducted in 3 L flasks filled with 0.6 L of medium in order to keep a 5:1 air–liquid ratio. Growth temperature was set at 37 °C and the culture was agitated at 140 rpm, in a rotary shaker incubator (model Minitron, Infors, Bottmingen, Switzerland). Experiments were performed on the medium containing per L: sucrose 17 ± 1 g, yeast extract 10 g, KH_2_PO_4_ 2 g, K_2_HPO_4_ 9.7 g/L, MnSO_4_·4H_2_O 0.1 g, and 1 mL of microelement solution (CaCl_2_ 2 g/L, MnSO_4_·4H_2_O 0.05 g/L, CuSO_4_·5H_2_O 0.019 g/L, ZnCl_2_ 0.046 g/L). pH was adjusted to 7.2 before strain inoculation. The medium also contained 5 μg/mL of chloramphenicol to avoid plasmid loss. Twenty ng/mL of nisin were added about 2–3 h after inoculating the culture to induce expression of recombinant proteins.

Experiments with the addition of 0.5 mM GalNAc, or 2 mM phosphatidylcholine after induction were also performed.

Determination of sucrose and acids produced during growth was performed by HPLC (UHPLC Dionex Ultimate 3000; Thermofisher) on a Alltech IOA-2000 column (250 mm × 6.5 mm ID). Three mL of broth collected at T_0_ and at the end of the process were centrifuged and supernatants were ultrafiltered on 3 kDa centricon devices (Millipore, Bedford, MA, USA) at 5000×*g* and the flow through was used for analyses. Runs were performed at 40 °C with 0.1% v/v sulphuric acid in water as mobile phase at a flow rate of 0.6 mL/min. Detection was performed via UV absorbance at 200 nm and refraction index (Shodex RI-101 detector, Max auto step 5.1 s, temperature 32 °C, rise time 1 s, polarity plus, record range 512 µRIU, integrator range 500 µRIU/UV).

### Extraction of intracellular polysaccharides

In order to extract intracellular chondroitin and HA, about 10 g of wet cells were resuspended in 20 mL of distilled water and autoclaved at 120 °C for 15 min. After centrifugation the supernatant was recovered and precipitated on ice with 4 volumes of cold ethanol 96% v/v, and stored at 4 °C o/n. The pellet, recovered after centrifugation was treated with 1 mg/mL DNase I (Applichem) in a buffer containing 100 mM Tris pH 7.5, 50 mM MgCl_2_ and 10 mM CaCl_2_ for 1 h at 37 °C and successively digested with 2.5 mg/mL of Protease K from *Aspergillus* (Sigma-Aldrich) for 2 h at 56 °C. A second precipitation was repeated on the sample o/n at 4 °C and the resulting pellet was dried and used for: (a) hydrodynamic characterization (b) quantification of uronic acids through carbazole assay (Bitter and Muir 1962) (c) determination of relative ratios of HA and chondroitin through high performance anion exchange chromatography with pulsed amperometric detection (HPAE-PAD) monosaccharide determinations after hydrolysis.

The powder obtained from the control sample was further purified to verify the production of chondroitin in *S. equi* subsp. *zooepidemicus*-pNZ8148*kfoAkfoC*, through structure determination by NMR analysis.

### Purification of intracellular chondroitin from *S. equi* subsp. *zooepidemicus*-pNZ8148*kfoAkfoC* by fast protein liquid chromatography (FPLC)

About 100 mg of powder were dissolved in 5 mL of buffer A (20 mM sodium acetate, 0.5 M sodium chloride, pH 7.4) and loaded on an anion exchange column (HiPrep Q Sepharose 16/10 HP, 1.6 × 10.0 cm, GE Healthcare, Milan, Italy), previously equilibrated with the same buffer, using an ÄKTA Explorer 100 purifier system (GE Healthcare, Milan, Italy), connected to the software UNICORN (GE Healthcare, Milan, Italy). The samples were eluted by applying a three steps gradient at 5, 10.5 and 100% of buffer B (20 mM sodium acetate, 3 M sodium chloride, pH 7.4) in 3 column volumes, at a flow rate of 3 mL/min and collected in 2 mL fractions. The chromatographic profiles were registered detecting the signal at 215 nm. The fractions containing a single peak were pooled together and each pool was loaded on a desalting column (HiPrep 26/10 Desalting, 2.6 × 10.0 cm, GE Healthcare, Milan, Italy) previously equilibrated with pure water. Elution was performed by using the same ÄKTA purifier system, at flow rate of 1 mL/min, in 1.5 column volumes; the chromatographic profiles were detected at 215 nm, the eluted peaks were collected and then freeze-dried (Christ Epsilon 2-6D, Martin Christ, Germany). After lyophilisation the peak fractions were analyzed by Size exclusion chromatography-triple detector array (SEC-TDA), NMR and hydrolysed for HPAE-PAD sugar nucleotide determinations.

### Precipitation of extracellular HA and chondroitin

After growth in shakeflask the broth was centrifuged at 6000×*g*, 4 °C for 30 min. The supernatant was precipitated with 1.8 volumes of cold ethanol (99.9% v/v) o/n at 4 °C to remove HA. The precipitate was recovered by centrifugation and vacuum dried at 40 °C for 18 h. The supernatant underwent a second precipitation, to recover chondroitin, with ethanol up to 4 volumes at 4 °C o/n and the recovered precipitate was dried at 40 °C under vacuum.

### Fermentation experiments

Fermentation experiments were carried out on a Biostat C plus reactor (Sartorius Stedim; Melsungen, Germany), with 1.6 L working volume. Agitation was provided by 1 rushton impeller and 2 paddle impellers, suitable for viscous liquid mixing. Fermentation medium contained per L: sucrose 70 g, MgSO_4_·7H_2_O 2 g, yeast extract 20 g, Na_2_HPO_4_·12H_2_O 2.23 g, K_2_SO_4_ 1.3 g, arginin 0.05 g and 2.5 mL of microelement solution. The preculture was performed in 1 L shakeflasks filled with 100 mL of medium, reported in the previous section, and grown on a rotary shaker at 200 rpm and 37 °C for 12–14 h. When pH dropped to 5 the flask culture was aseptically used to inoculate the fermenter: inoculum size was 5% of the volume of fermentation medium. A constant pH was maintained at 7.2 by automated addition of 6 M NaOH and 30% v/v sulfuric acid. Stirring and airflow rate were set at 200 rpm and 1.2/1.4 vvm for the duration of the experiment. The medium also contained 5 μg/mL of chloramphenicol and addition of 20 ng/mL of nisin was performed about 3 h after fermentation start. A pulse of sucrose re-establishing a concentration of 20 g/L in the broth was performed after 10 h of growth. At least four samples were withdrawn during the initial batch phase and at the end of the experiments to measure substrate consumption and production of lactic acid by HPLC as described in “Shakeflask experiments” section. The overall process lasted 18 h.

### Fermentation broth downstream processing to obtain heteropolysaccharides (HA and chondroitin)

The recovered fermentation broth was added with 5% v/v of trichloroacetic acid (from a 50% w/v solution) before centrifugation at 6000×*g* for 60 min. The pH of the clarified supernatant was adjusted to 6.5 ± 0.2 with 6 M NaOH. A first ultrafiltration (UF) step was performed on a tangential flow filtration system (Sartoflow Alpha crossflow system, Sartorius) by using a polyethersulfone membrane (0.1 m^2^) with a cut-off of 100 kDa. The supernatant was concentrated about eightfold, diafiltered with 2 volumes of bidistilled water and the retentate was collected. The cassette was washed with 100 mL of water and the recovered solution was added to the concentrated supernatant and precipitated with 1.8 volumes of cold ethanol (4 °C) o/n. The precipitate was vacuum dried at 40 °C for 18 h.

The 100 kDa permeate was further concentrated on UF polyethersulfone membrane (0.1 m^2^) with a cut-off of 10 kDa, concentrated tenfold and diafiltered with 300 mL of bidistilled water. The cassette was washed with 100 mL of purified water. Conductivity of the 10 kDa retentate and wash fraction was adjusted to about 15 mS/cm with NaCl before precipitation with 4 volumes of cold ethanol (4 °C) o/n. The recovered precipitate was vacuum dried at 40 °C for 18 h. The dried powder obtained from the 100 and 10 kDa retentate samples were analysed by SEC-TDA, HPAE-PAD and carbazole assay.

Intracellular polysaccharides were extracted as described in the previous paragraph (“Extraction of intracellular polysaccharides” section), and quantified as described above.

### Molecular weight determination

The molecular mass determinations of HA and chondroitin were carried out using the SEC-TDA 305 equipment by Viscotek (Malvern Instruments, Italy). Dried samples obtained after fermentation downstream treatments were dissolved in water and analysed whereas samples obtained from shakeflask experiments were dissolved in water and diafiltered with 2 volumes of bidistilled water on 3 kDa centrifugal filter devices (YM-10 Centricon, Millipore, Bedford, MA, USA) at 5000×*g* to remove salts and low molecular weight contaminants. Analyses were performed at concentrations ranging from 0.1 to 0.4 g/L for HA, and from 0.5 to 4 g/L for chondroitin, to have a column load for each sample (injection volume × sample concentration × intrinsic viscosity) of approximately 0.2 dL, and runs were performed at 40 °C with a running time of 50 min. The fragment molecular weight distribution, molecular size distribution, polydispersity, hydrodynamic radius, and intrinsic viscosity were determined as described by La Gatta et al. (2010) and Restaino et al. (2017).

### HPAE-PAD

The aminosugars were identified in extracellular and intracellular polysaccharide samples by using the high-performance chromatographic system equipped with a pulsed amperometric detector (PAD, Thermo Fisher Scientific, Italy) on an anion exchange column (Carbopac PA1, Thermo Fisher Scientific, Italy) as previously demonstrated by Marcellin et al. (2009). About 7 mg of powder (obtained after lyophilisation) were hydrolyzed in 1 M HCl for 18 h at 100 °C and analysed as described by Restaino et al. (2017).

### NMR analysis

NMR spectra were recorded with a Bruker DRX-600 (^1^H: 600 MHz, ^13^C: 150 MHz) instrument equipped with a cryo probe, in D_2_O (acetone as internal standard, ^1^H: (CH_3_)_2_CO at δ = 2.22 ppm; ^13^C: (CH_3_)_2_CO at δ = 31.5 ppm). The distorsionless enhancement by polarization transfer-heteronuclear single-quantum correlation (DEPT-HSQC) experiments were measured in the ^1^H-detected mode by single quantum coherence with proton decoupling in the ^13^C domain, by using data sets of 2048 × 256 points and typically 32 increments.

## Results

### Construction of recombinant *S. equi* subsp. *zooepidemicus*-pNZ8148*kfoAkfoC*

**Fig. 1:**
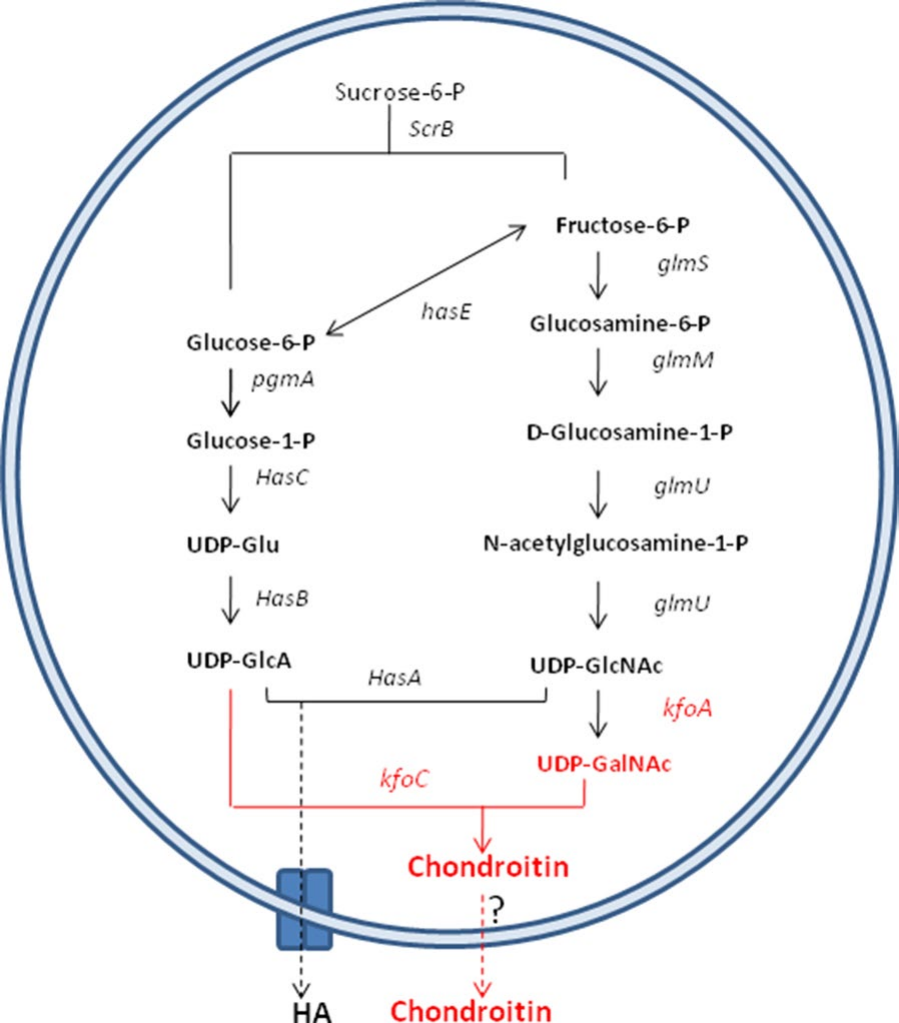
HA biosynthesis pathways in *Streptococcus*. The pathway was modified by the introduction of two foreign genes (indicated in *red*), *kfoA* coding for an epimerase and *kfoC* coding for chondroitin polymerase leading to the production of chondroitin in *S. equi* subsp. *zooepidemicus*

The pathways leading to HA production in *Streptococcus* spp. and the implemented changes performed in this work to obtain concurrent biosynthesis of chondroitin are shown in Fig. 1. PCR products were obtained utilizing *E. coli* K4 chromosomal DNA as a template, and specific oligonucleotide primers as indicated in the “Materials and methods” section. Sub-cloning for the construction of the recombinant plasmid pNZ8148*kfoAkfoC*, was performed in *L. lactis*. After electroporation recombinant cells were selected on medium containing chloramphenicol. Double digestions that yielded bands of the expected molecular weight, and gene sequence analyses confirmed the absence of mutations. *S. equi* subsp. *zooepidemicus BA06* was transformed by electroporation with pNZ8148*kfoAkfoC* and positive clones were selected on chloramphenicol.

### Production of chondroitin and HA in shake flask experiments

Growth of *S. equi* subsp. *zooepidemicus*-pNZ8148k*foAkfoC* was performed on medium containing sucrose and yeast extract as main C and N sources, and expression of recombinant genes was induced by the addition of nisin. The supernatant and the biomass were recovered and treated as reported in the “Materials and methods” section to establish the ratio of polysaccharides produced based on HPAE-PAD analyses of monomeric components (amino sugars and uronic acid); total polysaccharides produced were evaluated by carbazole assay based on uronic acid determination and results were consistent with those obtained by SEC-TDA. The recombinant strain demonstrated to consume about 9 g/L of sucrose within 18 h of growth, and to produce about 11 g/L of lactic acid. Strain performance was also evaluated in the presence of 0.5 mM GalNAc and 2 mM phosphatidylcholine. GalNAc administration did not alter biomass and lactic acid production, however sucrose consumption was 30% lower compared to control conditions, resulting in higher yields (Table 1, Additional file 2). Upon addition of phosphatidylcholine medium opalescence was observed; this did not allow absorbance determinations during strain cultivations. Lactic acid final concentration and sucrose consumption were not altered by the addition of this phospholipid.

**Table 1:** *S*. * equi* subsp. *zooepidemicus*-pNZ8148*kfoAkfoC* grown in shakeflasks on semidefined medium

Condition	OD_600_	Chondroitin extracellular (mg/L)	Chondroitin intracellular (mg/L)	Y_BC/Suc_ (mg/g)	HA extracellular (mg/L)	HA intracellular (mg/L)	Y_HA/Suc_ (mg/g)	Y_LA/Suc_ (g/g)
Nisin 20 ng/mL	8.2 ± 0.7	75 ± 8	13 ± 1 15%^a^	9.8 ± 0.3	412 ± 25	2.7 ± 0.2 0.6%^a^	47 ± 7	1.2 ± 0.1
Nisin 20 ng/mL, 0.5 mM GalNAc	8.7 ± 0.7	68 ± 6	24 ± 2 26%^a^	17.7 ± 1.5	366 ± 31	4.1 ± 0.4 1.1%^a^	71 ± 10	2.1 ± 0.2
Nisin 20 ng/mL, 2 mM phosphatidylcholine	nd	44 ± 4	16 ± 2 27%^a^	5.8 ± 0.85	428 ± 38	2.7 ± 0.3 0.6%^a^	42 ± 6	1.2 ± 0.1

Production of HA and chondroitin were analysed in the presence of inducer (nisin) alone or in combination with either GalNAc or phosphatidylcholine. Experiments lasted 18 h. Biomass and polymer concentrations indicated in the table were determined after 18 h of growth

*nd* not detectable

^a^Percentage of intracellular polymer on the total produced of each type (HA or chondroitin)

As reported in Table 1 about 90 mg/L of chondroitin in addition to 415 mg/L of HA were obtained from the recombinant strain after induction of *kfoC* and *kfoA* expression with nisin (control), demonstrating the proof of principle of the engineering strategy. Chondroitin concentration remained unchanged following medium supplementation with 0.5 mM GalNAc, whereas it was slightly lower in the presence of phosphatidylcholine. Also the concentration of HA was not significantly altered in all conditions. However, due to the lower concentration of sugar consumed with 0.5 mM GalNAc in the medium, the yield of chondroitin and HA with respect to starting sucrose (Y_BC/Suc_ and Y_HA/Suc_, respectively) were 80 and 54% higher compared to those obtained in control conditions (Table 1). The addition of a higher concentration of GalNAc (5 mM) improved HA production and decreased the amount of produced chondroitin (data not shown).

Extremely low fractions of HA (0.6–1%) on the total polysaccharide produced, were found inside *S. equi* subsp. *zooepidemicus*-pNZ8148k*foAkfoC* recombinant cells. Inversely, intracellular chondroitin amounts ranged from 15 to 27% of the total produced polysaccharide.

### Hydrodynamic characterization

The molecular weight (Mw) of the produced polysaccharides (HA and chondroitin) was assessed by SEC-TDA chromatography and results are reported in Table 2. The supernatants recovered from *S. equi* subsp. *zooepidemicus*-pNZ8148*kfoAkfoC* grown in shakeflasks, were initially precipitated with 1.8 volumes of ethanol in order to recover HA. As reported in Table 2A in all growth conditions this precipitate contained three peak distributions, the most abundant of which was that with higher molecular weight with values ranging from 51 to 59% (1st Peak, representativeness). Medium supplementation with 2 mM phosphatidylcholine increased the average Mw of this population by 18% compared to that obtained in control conditions; moreover, the analysis of Mw distributions of the subpopulations within this peak indicated a 10% larger subpopulation of molecules with a Mw above 2000 kDa. This result is also confirmed by the higher intrinsic viscosity (IV) of the sample recovered from recombinant cells growing in the presence of the above mentioned phosphatidylcholine. The presence of the latter also changed Mw distributions within the 3rd peak, in fact the amount of polymers with Mw > 50 kDa and with 10 kDa < Mw < 50 kDa were 34% lower and 36% higher in this condition, respectively (Table 2A).

**Table 2:** SEC-TDA analyses of polysaccharides produced by the recombinant strain of *S. equi* subsp. *zooepidemicus*-pNZ8148*kfoAkfoC* during growth in shakeflasks on semidefined medium

	*S. equi* subsp. *zooepidemicus*-pNZ8148*kfoAkfoC*		*S. equi* subsp. *zooepidemicus*-pNZ8148*kfoAkfoC* + 0.5 mM GalNAc		*S. equi* subsp. *zooepidemicus*-pNZ8148*kfoAkfoC* + 2 mM phosphatidylcholine	
*A: Analyses of polysaccharides precipitated with 1.8 volumes of ethanol*						
Ethanol precipitation (V)	1.8		1.8		1.8	
1st peak						
Mw (kDa)	1710	Mw distribution^a^: 31.7% > 2000 kDa	1709	Mw distribution^a^: 32.1% > 2000 kDa	1983	Mw distribution^a^: 42.5% > 2000 kDa
Mw/Mn	1.38		1.41		1.31	
IV (dL/g)	24.5		24.4		27.0	
Representativeness (%)	51.3		55.6		58.6	
2nd peak						
Mw (KDa)	720		241		511	
Mw/Mn	1.67		1.05		1.02	
IV (dL/g)	4.6		4.8		8.3	
Representativeness (%)	5.1		5.4		3.2	
3rd peak						
Mw (kDa)	53	Mw distribution^b^: 10 kDa < 48.7% < 50 kDa 51.3% > 50 kDa	41.8	Mw distribution^b^: 10 kDa < 48.3% < 50 kDa 51.7% > 50 kDa	70	Mw distribution^b^: 10 kDa < 66.0% < 50 kDa 34.0% > 50 kDa
Mw/Mn	1.55		1.24		1.22	
IV (dL/g)	1.0		1.0		1.0	
Representativeness (%)	28.9		24.9		21.3	
*B: Analyses of polysaccharides precipitated with 4 volumes of ethanol*						
Ethanol precipitation (V)	4		4		4	
1st peak						
Mw (KDa)	27.9		27.5		27.6	
Mw/Mn	1.07		1.06		1.05	
IV (dL/g)	0.1		0.2		0.1	
Representativeness (%)	22.4		24.3		14.8	
2nd peak						
Mw	19.8		19.2		19.3	
Mw/Mn	1.02		1.01		1.02	
IV (dL/g)	0.1		0.1		0.1	
Representativeness (%)	77.5		75.5		85.1	

*Mw/Mn* polymer polydispersity, *IV* intrinsic viscosity

^a^Molecular weight distribution of subpopulations found in the 1st peak indicating the percentage of molecules with a Mw > 2000 kDa

^b^Molecular weight distribution of subpopulations found in the 3rd peak

The supernatant was further precipitated with 4 volumes of ethanol to recover chondroitin and eventually residual low Mw HA. In all conditions two peaks with an average molecular weight of 27 and 19 kDa were found (Table 2B).

### Chondroitin purification and determination of chemical structure

Purification of chondroitin from the biomass was performed, after cell lysis and precipitation, by anion exchange chromatography. The three step gradient allowed the identification of four major fractions that subsequently underwent salt removal on a desalting column, hydrolysis, and HPAE-PAD for determination of monosaccharide components. The peak eluted at 5% v/v of buffer B showed the highest concentration of GalN and was further analysed by SEC-TDA and NMR.

**Fig. 2:**
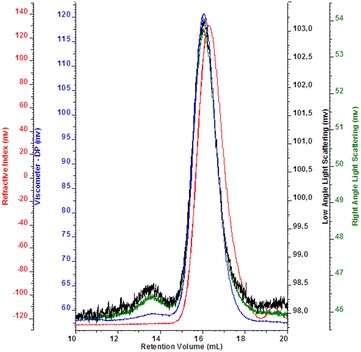
SEC-TDA chromatogram of intracellular chondroitin purified by ethanol precipitation, anion exchange and desalting chromatographies from *S. equi* subsp. *zooepidemicus*-pNZ8148*kfoAkfoC*. RI signal (*red*), viscometer signal (*blue*), right angle light scattering (*green*) and low angle light scattering (*black*)

**Fig. 3:**
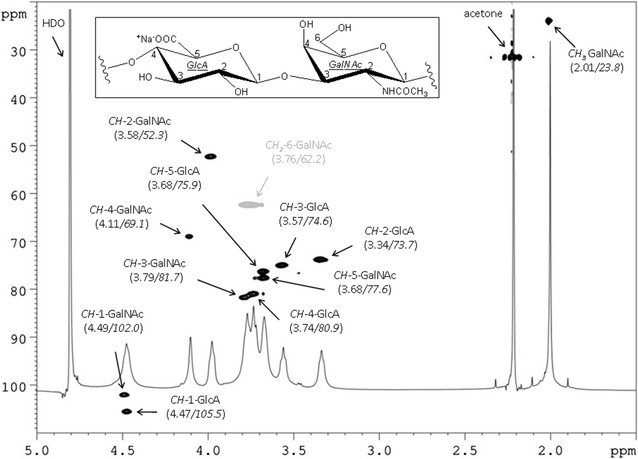
^1^H and DEPT-HSQC NMR (600 MHz, 298 K, D_2_O, acetone as internal standard) spectra of chondroitin polysaccharide from *S. equi* subsp. *zooepidemicus*-pNZ8148*kfoAkfoC* [chemical structure and numbering of chondroitin is shown in the *inset*; in the *parenthesis* below signal attributions are indicated the ^1^H and ^13^C (*in italic*) chemical shift values]

SEC-TDA analysis of the above indicated fraction showed a single peak (96% of representativeness) with a Mw of 28.4 kDa, a polydispersity index of 1.24 and an intrinsic viscosity equal to 1.09 as shown in the figure (Fig. 2). The chemical structure of the purified fraction was confirmed to be that of chondroitin by comparison of ^1^H and ^13^C NMR chemical shift data obtained by a DEPT-HSQC experiment (Fig. 3) with those reported in literature (Mucci et al. 2000).

This confirmed the production of chondroitin polysaccharide upon expression of the two *E. coli* K4 genes namely *kfoA*, that converted UDP-GlcNAc into UDP-GalNAc, and *kfoC*, coding for chondroitin polymerase, that is essential for polymer assembly, in *S. equi* subsp. *zooepidemicus BA06*.

### Batch experiments on 3 L-bioreactors

**Fig. 4:**
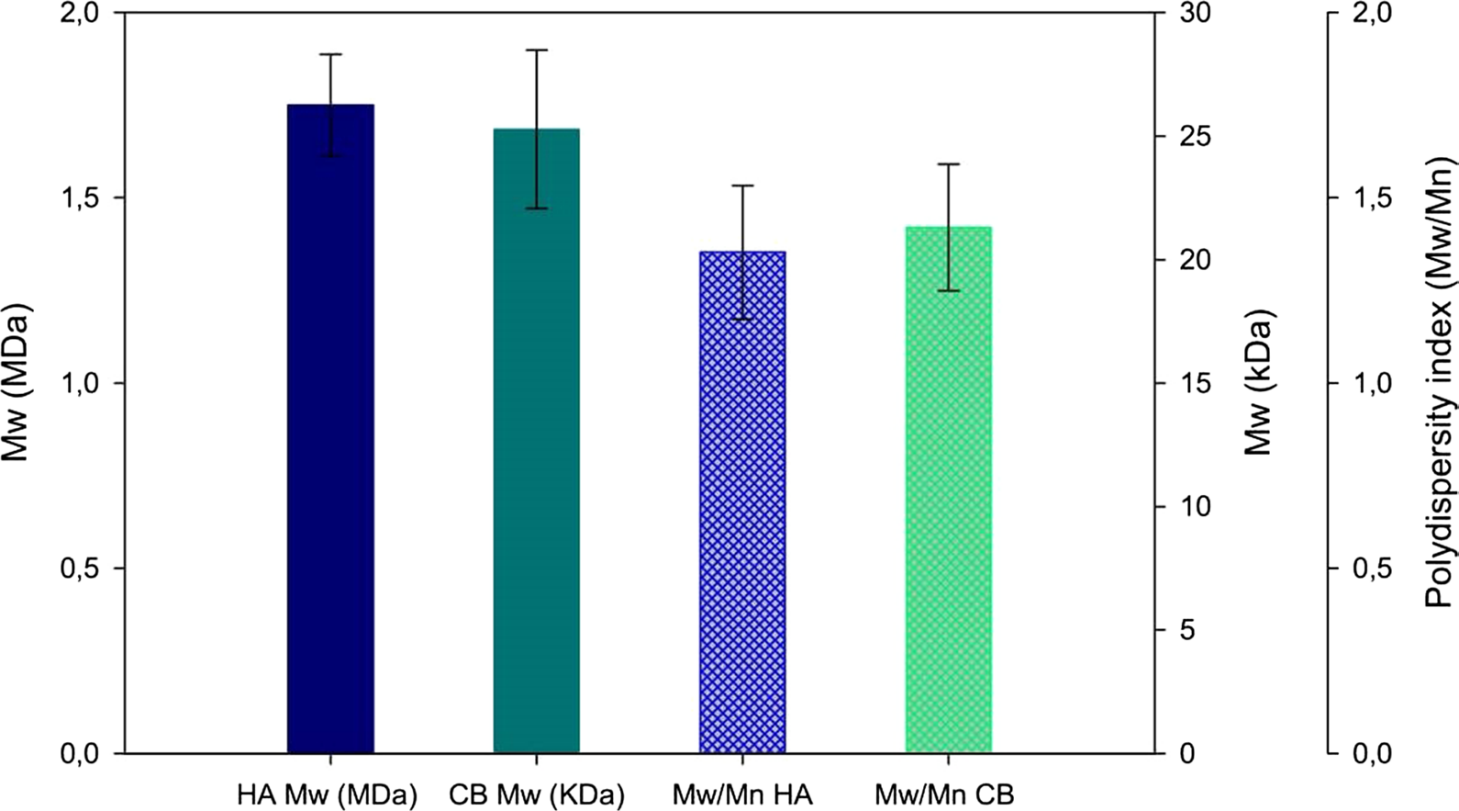
Average Mw and Mw/Mn obtained for HA and BC produced during batch fermentations on 3 L bioreactors

*S. equi* subsp. *zooepidemicus*-pNZ8148k*foAkfoC* was grown in a 3 L reactor on semidefined medium and expression of the foreign genes, *kfoC* and *kfoA*, was induced during exponential phase by the addition of nisin. The process lasted 18 h with a final concentration of biomass of about 8.34 ± 0.92 OD_600_ and a production of 50 ± 8 g/L of lactic acid (Table 3). The C source was not completely consumed, in fact, a residue of about 21 ± 2 g/L of sucrose was found in the medium at the end of the process (Additional file 3). The fermentation broth was treated with TCA to lower viscosity and to precipitate proteins in the medium and after centrifugation it was ultrafiltered/diafiltered on 100 kDa membranes to recover the high molecular weight fraction of exopolysaccharides; the permeate was concentrated and diafiltered on 10 kDa membranes. Both retentates were precipitated with ethanol in different proportion to recover HA and chondroitin enriched fractions, respectively (Additional file 4). An average production of 1.44 ± 0.36 and 0.22 ± 0.01 g/L of secreted HA and chondroitin, respectively, were found in the broth at the end of the process (Table 3; Additional file 3). Mw and polydispersity of the two produced polymers is reported in Fig. 4. The polysaccharide fraction extracted from the biomass after 18 h of growth consisted of about 0.021 and 0.082 g/L of HA and BC; data were normalized on the volume of recovered broth and on the concentration of wet biomass obtained at harvesting (Additional file 3).

**Table 3:** *S. equi* subsp. *zooepidemicus*-pNZ8148*kfoAkfoC* grown in 3 L bioreactors in batch conditions on semidefined medium

OD_600_	Y_xs_ (OD/g)	Y_LA/Suc_ (g/g)	HA extracellular (g/L)	BC extracellular (g/L)	HA intracellular (g/L)	BC intracellular (g/L)
8.34 ± 0.92	0.11 ± 0.01	0.62 ± 0.06	1.44 ± 0.36	0.22 ± 0.01	0.02 ± 0.00	0.08 ± 0.06

A pulse of 20 g/L of sucrose was performed after 10 h of growth. Results indicated in the table refer to data obtained at the end of the process after 18 h of growth

## Discussion

In the present work we reported the construction of a *S. equi* subsp. *zooepidemicus* recombinant strain that expresses two *E. coli* K4 genes, namely *kfoC* and *kfoA*, involved in the biosynthesis of chondroitin-like capsular polysaccharide. The aim of the study was to use a non-hemolytic and hyaluronidase negative microorganism, that already represents a consolidated industrial host for the production of HA, and endow it with the ability to additionally produce chondroitin, generating a cell factory for both biopolymers.

The difference between disaccharide units composing HA and chondroitin lies in the presence of either GlcNAc or GalNAc, respectively, bound to GlcA residues. Expression of *kfoA* encoding an epimerase that converts UDP-GlcNAc into UDP-GalNAc was not sufficient to redirect the pathways dedicated to HA biosynthesis in *S. equi* subsp. *zooepidemicus* to the production of chondroitin as well, probably due to the absence of a specific polymerase needed to bind nucleotide sugars and allow polymer assembly (data not shown). Concurrent expression of the *kfoC* encoded chondroitin polymerase, was necessary to produce chondroitin as confirmed by HPAE-PAD and NMR analyses. The recombinant strain *S. equi* subsp. *zooepidemicus*-pNZ8148*kfoAkfoC* co-produced about 400 and 90 mg/L of HA and chondroitin, respectively, in shakeflasks on semidefined medium containing sucrose and yeast extract as main C and N sources. A productivity of 23 mg/Lh of HA was obtained considering the entire process duration. The final titer and productivity of chondroitin was lower with respect to HA, but comparable to that obtained by growing a natural producer such as *E. coli* K4 in shakeflasks on semidefined medium (Cimini et al. 2010). While HA was completely secreted from recombinant cells with a residual intracellular concentration of about 1% of the total polysaccharide produced, a higher concentration of chondroitin, ranging between 15 and 27%, was found upon biomass extraction (Table 1), probably indicating a lower efficiency of the secretion system with the new polymer. Two aspects were further investigated in shakeflasks, namely (i) whether GalNAc supplementation could partially redirect fluxes towards chondroitin production (ii) whether addition of phosphatidylcholine would increase HA molecular weight.

The crystal structure of chondroitin polymerase suggested a higher binding affinity for UDP-GalNAc (Zanfardino et al. 2010) and, a simulated implementation of the UDP-GalNAc productive branch by glutamine addition boosted capsular polysaccharide production in recombinant *E. coli* K4 (Cimini et al. 2015). However, in the present work addition of GalNAc only resulted in a slight reduction of HA titers, leaving the production of chondroitin unchanged and a higher (80%) concentration of the latter inside the cells compared to that found in control conditions. Addition of growing concentrations of GalNAc up to 5 mM did not improve results leading, on the contrary, to higher HA final titers thereby also decreasing the amount of chondroitin produced (data not shown).

One of the most pursued research goals about HA production is to obtain HA with the highest possible molecular weight and several critical cultivation parameters such as pH, temperature, aeration conditions etc. were investigated so far (Liu et al. 2011). The ratio of UDP-GlcA and UDP-GlcNAc influences the molecular weight of HA produced by *Streptococcus* strains (Badle et al. 2014), however genetic changes made in this work did not exert such effect.

Sun et al. (2012) interestingly found that the presence of phosphatidylcholine in the growth medium directed more carbon to HA synthesis (production raised by 17%) by enhancing ATP production. It also increased the polymer molecular weight by 67% in controlled bioreactor experiments. Addition of the same phospholipid here did not enhance HA production. Nevertheless, an 18% higher average molecular weight was observed, thereby obtaining HA with average Mw of 1.98 kDa. In fact, interestingly, SEC-TDA showed that within the highest molecular weight peak (1st peak) obtained after the first precipitation step with ethanol, a 10% larger subpopulation of molecules with a Mw above 2000 kDa was found, compared to that observed in the absence of phosphatidylcholine (Table 2). Similar polydispersity (Mw/Mn) and higher IV compared to the control (27.0 vs 24.5 dL/g) further confirm data robustness. Moreover, the same sample showed, within the 3rd peak, a smaller amount of polymers with Mw > 50 kDa indicating a broader effect of phosphatidylcholine on the distribution of high and low molecular weight HA polymers.

SEC-TDA analyses of supernatants further precipitated with 4 volumes of ethanol indicated the presence of two peaks with an average molecular weight of 27 and 19 kDa. By comparing the relative abundance of the two peaks identified by SEC-TDA to results obtained by hydrolysis and HPAE-PAD, the two populations principally correspond to chondroitin together with residual polysaccharides from the medium and a lower HA contamination.

Constant air sparging and pH control led to the production of about 1.46 and 0.3 g/L of HA and BC respectively in 3 L bioreactors, in batch mode. Twenty-seven percent of chondroitin was retained inside the cells, whereas most HA was found in the extracellular environment (1.5% inside on the total produced), confirming shakeflask results. The polymers produced after 18 h of growth were characterized after 1/2 ultrafiltration steps and precipitation with differential ethanol percentages showing an average Mw of 1.75 ± 0.14 MDa and 25 ± 3 kDa for HA and BC, respectively.

Engineering of *S. equi* subsp. *zooepidemicus BA06*, a hyaluronidase and haemolysin-free natural producer of HA, was accomplished in this work. The strain was enriched by recombinant inducible expression of two *E. coli* K4 genes, *kfoA* and *kfoC*, that are involved in the biosynthesis of chondroitin-like polysaccharide. As proved by HPAE-PAD and NMR the recombinant strain produced both polymers with an HA:BC ratio equal to 5:1, for extracellular species, and 1:4/5 for intracellular species. To our knowledge this is the first study that provides an approach to the concurrent production of HA and chondroitin in a highly exploited industrial workhorse, that differently from other potentially harmful *Streptococcus* strains is non-hemolytic and hyaluronidase negative. This gives the opportunity to obtain both polymers from a single bioprocess; since the HA secretion apparatus is not as efficient for chondroitin release, biomass could be the source of a chondroitin rich polysaccharide fraction of low Mw (<40 kDa), whereas the supernatant the source of almost pure and high Mw HA.

## Additional files

**Additional file 1.** Sequence of *kfoA* and *kfoC* genes amplified from *E. coli* K4 and cloned in pNZ8148; Map of pNZ8148*kfoAkfoC.*

**Additional file 2.** Complete data of shake flask experiments of *S. equi* subsp. *zooepidemicus*-pNZ8148*kfoAkfoC* grown in shakeflasks on semidefined medium in the presence of inducer (nisin) alone or in combination with either GalNAc or phosphatidylcholine. Three experiments were performed in each condition. Mean and sd resulting from these experiments are reported in Table 1.

**Additional file 3.** Triplicate experiments regarding growth of *S. equi* subsp. *zooepidemicus*-pNZ8148*kfoAkfoC* in 3 L bioreactors in batch conditions. SEC-TDA results for the determination of Mw and polydispersity of HA and BC are also reported. Mean and sd values are reported in Table 3 and Fig. 4.

**Additional file 4.** Downstream processing of broths recovered from growth of *S. equi* subsp. *zooepidemicus*-pNZ8148*kfoAkfoC* on 3 L bioreactors.

## Abbreviations

CS: chondroitin sulphate
BC: biotechnological chondroitin
HA: hyaluronic acid
GlcA: glucuronic acid
GalNAc: *N*-acetylgalactosamine
GlcNAc: *N*-acetylglucosamine
