# Supplementary material for: Engineering S. equi subsp. zooepidemicus towards concurrent production of hyaluronic acid and chondroitin biopolymers of biomedical interest
Source: AMB Express. 2017 Mar 14;7:61. doi: 10.1186/s13568-017-0364-7 (PMC5350083; doi:10.1186/s13568-017-0364-7)
Supplement: Supplementary file 1 — Additional file 1. Sequence of kfoA and kfoC genes amplified from E. coli K4 and cloned in pNZ8148; Map of pNZ8148kfoAkfoC. [file 13568_2017_364_MOESM1_ESM.docx]

**Additional File 1**

**Engineering *S. equi* subs. *zooepidemicus* towards concurrent production of hyaluronic acid and chondroitin biopolymers of biomedical interest**

Donatella Cimini^a*^, Ileana Dello Iacono^a^, Elisabetta Carlino^a^, Rosario Finamore^a^, Odile F. Restaino^a^, Paola Diana^a^, Emiliano Bedini^b^, Chiara Schiraldi^a^*.

^a^ University of Campania Luigi Vanvitelli (ex Second University of Naples), Department of Experimental Medicine, Via de Crecchio 7, 80138, Naples, Italy.

^b^Department of Chemical Sciences, University of Naples "Federico II", Complesso Universitario Monte S. Angelo, via Cinthia, 4, 80126 Naples, Italy^.^

****Corresponding author***

**Sequences of the *kfoA* and *kfoC* genes amplified from the genome of *E.coli* K4.**

***kfoA***

atgaatatattagttacaggtggagcaggctatattggctcgcatactagtttatgtcttctgaataaaggttacaatgttgtaatcattgacaacttaattaattcatcttgcgagagcattcgaaggattgaattaatagctaaaaaaaaagttactttctatgagttgaacatcaacaatgaaaaagaagttaatcaaattctaaaaaaacacaaatttgattgtataatgcattttgccggtgcaaagtctgttgctgaatctttaataaaacccattttttattatgataataatgtttcagggacgttgcaattaattaattgcgctataaaaaacgatgtggctaattttatttttagctcttctgcaacggtttatggtgaaagcaaaataatgcctgttacagaagattgccatataggaggaacattaaatccatatggtacatcaaagtatatatcagaattgatgattagagatattgcaaaaaaatatagcgatactaattttttgtgtctgagatattttaacccaacaggtgctcacgagtcgggaatgatcggtgaaagtcccgctgatataccaagcaatttagttccttatatattacaagttgctatgggtaaactagaaaaacttatggtgtttgggggggattaccctacaaaggatggaaccggtgttcgtgattatatacacgtaatggatttagcggaagggcatgtggctgctttatcttaccttttccgtgataataacactaattatcatgtttttaatttaggtactggtaaaggatattctgttttagagctggtttctacctttgaaaaaatatctggggttagaattccatatgaaattgtttcgagaagagatggggatattgctgaaagttggtcatcaccagaaaaagcaaataagtatctcaattggaaagctaaaagggaattggaaacaatgcttgaggatgcctggcgctggcaaatgaaaaacccaaatggttatatttaa

***kfoC***

atgagtattcttaatcaagcaataaatttatataaaaacaaaaattatcgccaagctttatctctttttgagaaggttgctgaaatttatgatgttagttgggtcgaagcaaatataaaattatgccaaaccgcactcaatctttctgaagaagttgataagttaaatcgtaaagctgttattgatattgatgcagcaacaaaaataatgtgttctaacgccaaagcaattagtctgaacgaggttgaaaaaaatgaaataataagcaaataccgagaaataaccgcaaagaaatcagaacgggcggagttaaaggaagtcgaacccattcctttagattggcctagtgatttaactttaccgccgttacctgagagcacaaacgattatgtttgggcggggaaaagaaaagagcttgatgattatccaagaaaacagttaatcattgacgggcttagtattgtaattcctacatataatcgagcaaaaatacttgcaattacacttgcttgtctttgtaaccaaaagaccatatacgactatgaagttattgttgccgatgatggaagtaaagaaaatattgaagaaatagtaagagaatttgaaagtttattaaatataaaatatgtacgtcagaaggattatggatatcaactgtgtgctgttagaaatcttgggcttagggctgcaaagtataattatgttgcaattctggattgtgatatggctccgaacccactatgggttcagtcatatatggaactattagcggtggacgataatgttgctctaattggccctagaaaatatatagatacaagcaagcatacatatttagatttcctttcccaaaaatcactaataaatgaaattcctgaaatcattactaataatcaggttgcaggcaaggttgagcaaaacaaatcagttgactggcgaatagaacatttcaaaaataccgataatctaagattatgcaacacaccatttcgattttttagcggaggtaatgtcgcttttgcgaaaaaatggcttttccgtgcaggatggtttgatgaagagtttacgcattgggggggggaggataatgagtttggatatcgtctctacagagaaggatgttactttcggtctgttgaaggagcaatggcatatcatcaagaaccacccgggaaagaaaacgagacggatcgtgcggcagggaaaaatattactgttcaattgttacagcaaaaagttccttatttctatagaaaaaaagaaaaaatagaatccgcgacattaaaaagagtaccactagtatctatatatattcccgcctataactgctctaaatatattgttcgttgtgttgaaagcgcccttaatcagacaataactgacttagaagtatgcatatgcgatgatggttccacagatgatacattgcggattcttcaggagcattatgcaaaccatcctcgagttcgttttatttcacaaaaaaacaaaggaattggttcagcatctaatacagcagttagattgtgtcggggattctatataggtcagttagactctgatgactttcttgaaccagatgctgttgaactatgtctagatgaatttagaaaagatctatcattggcatgtgtttatacaactaaccgtaatatagatcgtgaaggtaatttgatatcaaatggctataattggcccatttattcgcgagaaaaacttactagtgcaatgatatgtcatcatttcaggatgttcacagcaagagcatggaacctaactgaaggtttcaacgaatcgatcagcaacgcagttgattacgatatgtatttaaaacttagtgaagttggaccgttcaagcatataaacaaaatttgttataatcgcgtattgcatggtgaaaatacgtctataaaaaagttggatattcaaaaggaaaatcattttaaagttgttaacgaatcattaagtaggctaggcataaaaaaatataaatattcaccattaactaatttgaatgaatgtagaaaatatacctgggaaaaaatagagaatgatttataa

**Map of the recombinant plasmid pNZ8148*kfoAkfoC***

**
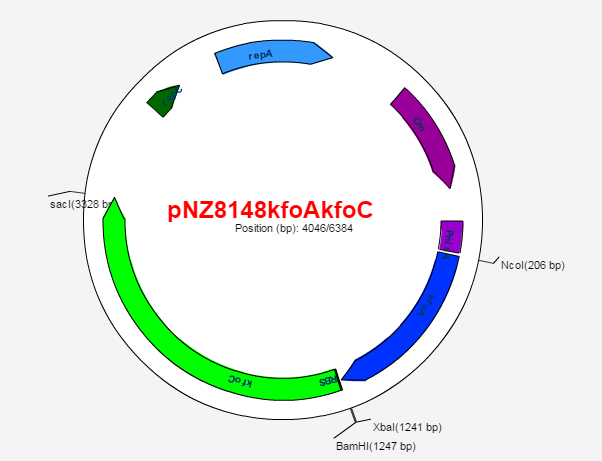
**
