# Supplementary material for: Engineering S. equi subsp. zooepidemicus towards concurrent production of hyaluronic acid and chondroitin biopolymers of biomedical interest
Source: AMB Express. 2017 Mar 14;7:61. doi: 10.1186/s13568-017-0364-7 (PMC5350083; doi:10.1186/s13568-017-0364-7)
Supplement: Supplementary file 4 — Additional file 4. Downstream processing of broths recovered from growth of S. equi subsp. zooepidemicus-pNZ8148kfoAkfoC on 3 L bioreactors. [file 13568_2017_364_MOESM4_ESM.docx]

**Supplementary file 4**

**Engineering *S. equi* subs. *zooepidemicus* towards concurrent production of hyaluronic acid and chondroitin biopolymers of biomedical interest**

Donatella Cimini^a*^, Ileana Dello Iacono^a^, Elisabetta Carlino^a^, Rosario Finamore^a^, Odile F. Restaino^a^, Paola Diana^a^, Emiliano Bedini^b^, Chiara Schiraldi^a^*.

^a^ University of Campania Luigi Vanvitelli (ex Second University of Naples), Department of Experimental Medicine, Via de Crecchio 7, 80138, Naples, Italy.

^b^Department of Chemical Sciences, University of Naples "Federico II", Complesso Universitario Monte S. Angelo, via Cinthia, 4, 80126 Naples, Italy^.^

**Downstream processing of broths recovered from growth of *S. equi* subs. *zooepidemicus*-pNZ8148*kfoAkfoC* on 3L bioreactors.**

After addition of TCA and centrifugation of the broth the recovered supernatant was ultrafiltered on 100 kDa; the recovered permeate was ultrafiltered on 10 kDa membranes as reported in the Materials and Methods section, and the recovered concentrated retentates (>100 kDa and >10 kDa) were precipitated with 1.8 and 4 volumes of cold ethanol (4°C), respectively.

The distribution of HA and CB following UF on 100 kDa and 10 kDa membranes and precipitation is reported in the following table:

|  | **Mw>100kDa (%)** | | **<10kDaMw<100kDa (%)** | |
| --- | --- | --- | --- | --- |
|  | **HA** | **CB** | **HA** | **CB** |
| **Batch 1** | 100 | 0 | 20 | 80 |
| **Batch 2** | 98 | 2 | 45 | 55 |
| **Batch 3** | 97 | 3 | 40 | 60 |

Fig. 1- SEC-TDA chromatogram exemplifying the analysis of the recovered supernatant ultrafiltered on 100 kDa membranes. RI signal (red), Viscometer signal (blue), Right Angle Light Scattering (green) and Low Angle Light Scattering (black).

Fig. 2- SEC-TDA chromatogram exemplifying the analysis of the 100 kDa permeate ultrafiltered on 10 kDa membranes. RI signal (red), Viscometer signal (blue), Right Angle Light Scattering (green) and Low Angle Light Scattering (black).
